# Dietary Inflammatory Nutrients and Esophageal Squamous Cell Carcinoma Risk: A Case-Control Study

**DOI:** 10.3390/nu14235179

**Published:** 2022-12-05

**Authors:** Shanshan Li, Joshua Ye, Zheng Lin, Zhifeng Lin, Xuwei Tang, Wenqing Rao, Zhijian Hu

**Affiliations:** 1 Department of Epidemiology and Health Statistics, School of Public Health, Fujian Medical University, Fuzhou 350122, China; 2 The Quarry Lane School, Dublin, CA 94568, USA; 3 Key Laboratory of Ministry of Education for Gastrointestinal Cancer, Fujian Medical University, Fuzhou 350122, China

**Keywords:** diet, nutrient, esophageal squamous cell carcinoma, inflammation

## Abstract

We conducted a case-control study (532 cases and 532 control) in Chinese adults to investigate the independent and interactive effects of dietary nutrients (pro- or anti-inflammation) on Esophageal Squamous Cell Carcinoma (ESCC) risk. Dietary data were collected using a food questionnaire survey that included 171 items. Two algorithms, the Least Absolute Shrinkage and Selector Operation (LASSO) and Bayesian Kernel Machine Regression (BKMR) were employed to select indicators and evaluate the interactive effect of nutrients’ mixture on ESCC risk. Thirteen nutrients were selected, including three pro-inflammatory nutrients (protein, fat and carbohydrate) and ten anti-inflammatory nutrients (fiber, Vitamin A, riboflavin, niacin, Vitamin C, Fe, Se, MUFA, n-3 PUFA and n-6 PUFA). Single-exposure effects of fat, carbohydrate and fiber significantly contributed to ESCC risk. The pro-inflammatory nutrients’ submodel discovered that the combined effect was statistically associated with increased ESCC risk. In addition, a higher fat level was significantly associated with ESCC risk. On the contrary, for fiber and riboflavin, the anti-inflammatory nutrients’ submodel delineated a significant negative effect on the risk of ESCC. Our result implies that dietary nutrients and their inflammatory traits significantly impacted ESCC occurrence. Additional studies are warranted to verify our findings.

## 1. Introduction

Esophageal cancer (EC) is the seventh-most common malignancy of the upper gastrointestinal tract worldwide, with aggressive potency and a heavy burden of 570,000 new cases occurring annually based on global cancer 2020 (http://globocan) (accessed on 23 December 2021) [1,2]. As a deadly histological subtype, esophageal squamous cell carcinoma (ESCC) is characterized by a late-stage diagnosis and challenging clinical management [3]. Hence, investigating risk factors associated with ESCC has significant public and clinical implications for achieving an early prevention and diagnosis. 

Chronic inflammation is a known risk factor for ESCC [4,5]. Cyclooxygenase, a target of NSAIDs, and its downstream bioactive lipid products might provide evidence of the link between inflammation and ESCC [6]. In addition, activation of the immune system and chronic inflammation—induced by heat injury, further strengthened genetic alterations and intercellular signaling pathways, including the nuclear factor kappa-light chain enhancer for activated B cells—signal the transduction activator for transcription-3 and hypoxia-inducible factor 1α to regulate the malignant progression of cancer cells [7]. Notably, overexpression and activation of sphingosine kinase 1 (SphK1) promoted and enhanced the development and progression of ESCC. Sphingosine 1-phosphate (S1P), a product of SphK1, led to various inflammatory reactions, including lymphocyte transport. A high fat and sugar diet, unbalanced intestinal flora and obesity are associated with inflammation activation and SphK/S1P/S1P receptor signal transduction in various gastrointestinal pathologies (including cancer) [8]. 

Accumulating evidence suggested that inflammatory effects of dietary components and embedded nutrients might be involved in esophageal tumorigenesis and progression [9,10]. Fruits and vegetables containing abundant β-carotene, fiber and other antioxidants have been hypothesized to possess a protective prospect [11]. In contrast, salted consumption redundant in sodium and meat diets predominant in fat seemed to expose a dismal effect on ESCC by the postulated mechanism, which was thought to accelerate carcinogenesis through systemic inflammation and various cellular processes interference. In real life scenarios, the regular practice we employed was syncretism with various foodstuffs instead of eating particular foods or nutrients alone, which might lead to countless permutations and combinations of the health effects of food components. Therefore, merely from the perspective of a single nutrient or a single food group in replacement of overall diets to grope for the relationship between improper dietary compositions and ESCC risk, this cannot tangibly reflect actual intake, comprehensive effect and potential interaction among food and within nutrients. The paucity of integrative analyses concentrating on the relationship between multiple nutrients and ESCC highlights key future research priorities.

The dietary inflammatory index (DII) is an indicator synthesizing the intake of dietary constituents and evaluating associations between diets with well-known inflammatory markers [12]. DII specifies inflammatory scores for each food or nutrient by way of the extensive literature and categorizes individuals’ diets according to their inflammatory potential on a continuum from maximally pro-inflammatory to maximally anti-inflammatory. Based on the objectively and reliably inflammatory effect score for each nutrient or food, the main focus of this study (i.e., dietary nutrients), was divided into pro-inflammatory and anti-inflammatory. Current interests tend to be given to the single effect of singular nutrients or the whole effect of comprehensive indicators, with the default of graphical presentation and multi-level consideration about the interactions between nutrients. In addition to the exploration of the overall effect reflected by DII, we also want to explicit single meticulousness influence and specify detailed interactions among the inflammatory nutrients’ collocation on ESCC risk and then further explain and complement the possible mechanism implied by the whole effect. 

The Least Absolute Shrinkage and Selector Operation (LASSO) [13] could identify the most relevant nutrient variables, while the Bayesian Kernel Machine Regression (BKMR) [14], a relatively new algorithm, could further address the linear or non-linear interrelation of multiple exposed variables (dietary nutrients with distinct inflammatory potency) in a specific outcome as dependent variables (ESCC carcinogenesis). BKMR models in this study could estimate the mixture effects, detect the interactions among individual nutrients and explore their relationship with ESCC risks. This implicated the relationship between dietary nutrients and ESCC that might not be simply predicted by DII. The exploration of the interaction and other effects of nutrients by BKMR can be used as a favorable supplement to DII so as to more holistically evaluate the influence of inflammatory diets on ESCC and preliminarily provide causal inference clues for the follow-up mechanism research [15,16,17]. Consequently, this study aims to do the following: (1) investigate the association of multiple dietary nutrients’ (cataloged into pro- and anti-inflammation) co-exposure with ESCC risk; (2) evaluate the impact of single-effect and interaction within different inflammatory levels of nutrients on ESCC risk through implementing a BKMR model, providing preliminary causal inferences in diet-related carcinogenesis and a reference base for the nutritional prevention of ESCC. In the current study, we hypothesized that the data-driven LASSO and BKMR algorithms would supplement traditional models of the associations of single nutrients/indices in elucidating the relationship between dietary inflammatory nutrients and the ESCC risk. To be compared with conventional studies [18,19,20], we also assessed the association of DII calculated by nutrients with ESCC risk.

## 2. Materials and Methods

### 2.1. Study Design and Participants

This study was designed as a case-control evaluation. The case group was newly diagnosed ESCC patients with detailed clinicopathological data collected from the First Affiliated Hospital of Fujian Medical University and Fujian Provincial Cancer Hospital (through 2021), while the carcinoma-free control group was age- and sex-matched healthy population randomly enrolled from community or village registers in Fujian Province at the same period. All of the subjects in this study were self-reported as no special diet behaviors, such as long-term diet modification and major diet change in the past five years. This project belonged to the sub-project of the special disease cohort of EC in Fujian Province. The study was approved by the Ethics Committee of Fujian Medical University (approval no. 201495). All study subjects provided written informed consent.

Cases were eligibly complying with the inclusion criteria below: ① Incident cases of ESCC confirmed by X-ray, endoscopy or pathology after baseline enrollment according to the International Classification of Disease for Oncology (ICD-O), 10th edition; ② The average daily energy intake is greater than 700 kcal and less than 4200 kcal for males while the average daily energy intake is greater than 500 kcal and less than 3500 kcal for females; ③ Signed informed consent forms and completely qualified questionnaire survey data were equipped. Moreover, subjects were excluded as unreliable according to the exclusion criteria below: ① failure to complete or cooperate with the questionnaire survey with confidence or reliability; ② recurrent, secondary or prevalent ESCC cases were diagnosed by pathology; ③ having or ever had suffered from severe diseases such as malignancy or immunological defects.

**Figure 1:**
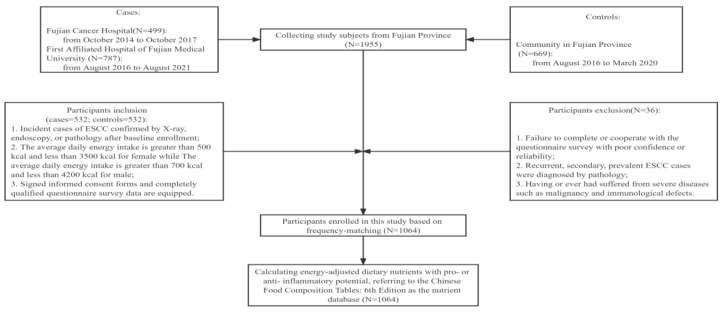
Flowchart of Subject Inclusion and Exclusion Criteria.

Controls were qualified and recruited according to the following inclusion criteria: ① without history of any malignant disease or other diseases that may distinctly change the diet, such as diabetes and arthrolithiasis; ② the average daily energy intake is greater than 700 kcal and less than 4200 kcal for males while the average daily energy intake is greater than 500 kcal and less than 3500 kcal for females; ③ signed informed consent forms and completely qualified questionnaire survey data are equipped. Moreover, controls were excluded in the light of the following exclusion criteria: ① failure to complete or cooperate with the questionnaire survey with confidence or reliability; ② having or ever had suffered from severe diseases such as malignancy or immunological defects. Of the 1,955 participants through 2021, we identified 1064 subjects with dietary data. After excluding participants who suffered from severe diseases, or for whom there was incomplete information, 1064 adult subjects were finally included in the study (Figure 1). 

### 2.2. Data Collection

All the survey data were collected through face-to-face interviews using constructed and standardized questionnaires by trained personnel. Sociodemographic characteristics (e.g., gender, age, education, occupation, income), behavioral habituation (e.g., tobacco smoking, alcohol drinking, tea consumption) and dietary information (e.g., frequency of hot food intake, eating speed per meal) were ascertained via questionnaire.

### 2.3. Dietary Measurements

Various food intakes commonly consumed in Southeast China were assessed using a 171-item semi-quantitative food-frequency questionnaire (FFQ), classified into five major categories: (1) cereals, 14 items; (2) plant-based foods, 82 items; (3) animal-based foods, 49 items; (4) beans, peas, legumes and their products, 8 items; (5) beverages, desserts and nuts, 18 items. For each food, the subjects reported the frequency of consumption, which was grouped into four classes including <1 time/month, 1–3 times/month, 2–6 times/week, ≥1 time/day, during one year before the diagnosis for cases or before the interview for controls. The interviewers utilized multifarious food molds in the dietary survey to assist subjects in recalling the specific consumption amounts more accurately.

### 2.4. Inflammatory Nutrients

We captured 23 of the 45 food parameters, which composed DII algorithm and were quantified in inflammatory potential. In this study, energy (kcal), protein (g), carbohydrate (g), fiber (g), cholesterol (mg), fat (g), saturated fatty acid (SFA) (g), monounsaturated fatty acid (MUFA) (g), polyunsaturated fatty acid (PUFA) (g), β-carotene (μg), Vitamin A (RE), thiamine (mg), riboflavin (mg), Vitamin C (mg), Vitamin E (mg), niacin (mg), Ferrum (Fe) (mg), magnesium (Mg) (mg), selenium (Se) (μg), zinc (Zn) (mg), n-3 PUFA (g) and n-6 long-chain PUFA (n-6 PUFA) (g) contents were obtained from FFQ data. Nutrient intakes were calculated through multiplying the nutrient-content of each daily food per 100 g by the intake frequency and summed across all food items, referring to the China Food Composition Tables: 6th edition. In addition, the daily average intake of alcohol (g) was directly obtained from the survey data based on the China Food Composition Tables: 6th edition. 

In the analysis of dietary nutrients and ESCC risk (i.e., LASSO and BKMR models), all nutrients were adjusted to the total energy take by estimating the energy-adjusted intakes of each food item using the FFQ [21] and classified into pro- and anti-inflammatory predictors according to the representative and standardized database [12] rooted in 11 countries around the world, which evaluated the comprehensive analysis of 929 articles on diet and body inflammatory indicators published during 1950–2007 with sufficient literature foundation and provided a credible inflammatory effect score for each nutrient or food with reliable validity. To explore the true correlation between dietary nutrients and ESCC in excluding the confounding effect of total energy intake from dietary components, the nutrients were calculated per 1000 kcals of food consumed.

The process of development and calculation of the DII have been described in detail previously [12]. The calculation of DII was accomplished by deducting population-estimated average daily intake and divided by standard deviation. To minimize the influence of the “right deviation”, the obtained Z-value was converted into percentile to centralize on 0 by doubling the proportion and subtracting 1. Eventually, the sum of total DII score was calculated to multiply by the corresponding inflammatory effect score of each nutrient or food, and all figures were added together. To control for the potential influence of energy intake, the DII was calculated per 1000 kcals of food consumed based on the energy-density model (during the calculation of DII, nutrients were not energy-adjusted). The values of single inflammatory effect score vary between −1 (maximum anti-inflammation) and +1 (maximum pro-inflammation). For the purposes of this paper, DII score was divided into quartiles in accordance with cut points derived from its distribution among controls, with quartile 1 as the referent group. Finally, there were 22 dietary nutrients included in our study, which consisted of 16 nutrients with anti-inflammatory effects and six nutrients with -pro-inflammatory effect.

### 2.5. Covariates

Basic information comprised of gender (male, female), age (<60, ≥60), education (Illiteracy/primary school, junior high school/above), income (<CNY 2000/month, CNY 2000–5000/month, >CNY 5000/month), occupation (farmer, worker, individual business, other), tobacco smoking (no, yes), drinking intensity (no, mild, moderate, heavy), tea consumption (never/seldom, often), eating speed per each meal (normal, quick, slow), hot food (no, occasionally, often).

In this study, current alcohol drinker was defined as drinking at least once a week on average and the drinking amount was equivalent to at least 50 mL alcohol contents lasting for six months at fewest [22]. The intensity of alcohol consumption was categorized based on the daily intake of ethanol. Among them, daily alcohol consumption less than 12.5 referred to “mild drinking”, 12.5–50 referred to “moderate drinking” and >50 referred to “heavy drinking” [23]. According to the China Food Composition Tables: 6th edition, the respective ethanol contents of various alcohol drinks were listed as below. For 100 mL of alcoholic beverages, high-degree liquor (≥40 degree) is 52%, low-degreeiquor (<40 degree) is 38%, yellow rice or millet wine is 18%, fruit and red wine is 10%, and beer is 4%. Smoker was defined as smoking at a minimum of one cigarette per day for six months at lowest [24], and tea drinker was defined as drinking tea once per week at any rate on average for leastwise six months [25]. 

### 2.6. Least Absolute Shrinkage and Selection Operator Regression (LASSO)

**Figure 2:**
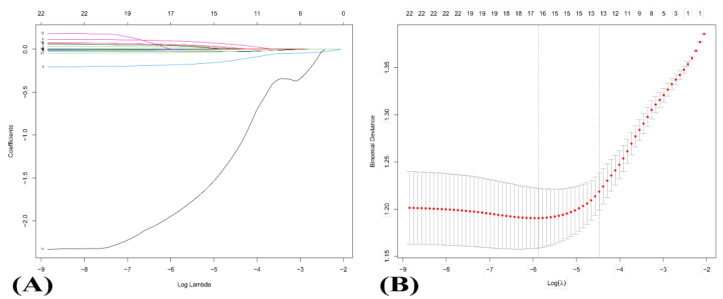
Variable selection using the LASSO logistic regression model. (**A**) LASSO coefficients of 22 candidate variables. The different color lines stand for trajectories for various variables at increasingly higher levels of λ. (**B**) The optimal penalization coefficient (λ) identification in the LASSO model was achieved by 10−fold cross−validation and the minimum criterion. The left vertical line represents the minimum error, and the right vertical line represents the cross−validated error within one standard error of the minimum. The red dotted line reflects changes of the mean squared error, defined as the sum of variance and squared bias, at different levels of λ. Abbreviation: LASSO, least absolute shrinkage and selection operator.

LASSO regression [13], an effective methodology for high-dimensional surface of predictors (22 pro- or anti-inflammatory nutrients), compressed function estimation and constructed a penalty function in the interest of variable selection, turning on reducing coefficients of variable sets. Moreover, it could avoid the malpractice resulting from ordinary least squares estimation, such as overfitting and multicollinearity. In this study, 13 inflammatory nutrients (Figure 2) were picked out with ten-fold cross-validation and one standard error of the minimum logarithmic transformation lambda value to be evaluated in the subsequent BKMR models.

### 2.7. Bayesian Kernel Machine Regression

BKMR [14], a relatively new statistical approach, addressed the interrelation of multiple exposed variables (dietary nutrients with distinct inflammatory potency) in a specific outcome as dependent variables (ESCC carcinogenesis). BKMR model utilized Gaussian kernel function to clarify the non-linear and non-additive exposure–response relationship through iterative regression, combining the Bayesian algorithm and statistical methodology. The main idea behind BKMR captured a wide range of underlying functional forms, expressed as the formula below:

$$g\left(\mu_{m}\right)=\exp\left\{-\sum_{m=1}^{M}r_{m}{\left(z_{m}-z_{m}^{\prime }\right)}^{2}\right\}+{\beta x}_{m}+\varepsilon_{m},m=1,\ldots ,n.$$

Here, g_i_ was a monotonic link function, μ_i_ = E(Y_i_); z and z’ represented vectors of predictor variables (22 inflammatory nutrients) for two different individuals; m referred to the study subject; r_m_ ≥ 0 denoted the tuning parameter that controlled the function smoothness of the exposure variables z_m_; x was a vector of covariates assumed to have had a linear relationship with ESCC (β as the corresponding vector of coefficients); *ε*_i_ referred to residual. Intuitively, the kernel function shrunk the estimated health effects of two individuals with similar nutrients profiles toward each other as the exposure–response function.

### 2.8. Statistical Analysis

Categorical variables were expressed as the count (N) and proportion (percentage). Normally distributed continuous variables were presented as means and standard deviations, whereas non-normally distributed data were presented as median with interquartile range. Chi-square and Mann–Whitney U tests were applied to compare basic characteristics between ESCC cases and controls for qualitative and quantitative data separately.

Given different inflammatory effects, DII-related food parameters were divided into pro-inflammatory and anti-inflammatory nutrients and then were selected by LASSO regression. In this study, three BKMR models were run by 10,000 iterations through the Markov Chain Monte Carlo algorithm, which contained three pro-inflammatory nutrients (protein, fat, carbohydrate) or/and ten anti-inflammatory nutrients (fiber, Vitamin A, riboflavin, niacin, Vitamin C, Fe, Se, MUFA, n-3 PUFA, n-6 PUFA). We regraded the concentration of each nutrient via energy-density adjustment as a continuous variable. We fitted the BKMR model to investigate the interaction, the combination, and the single effects of nutrient co-exposure on ESCC (dichotomous outcome). Potential confounding variables, including gender, age, education, income, occupation, tobacco smoking, drinking intensity, tea consumption, eating speed per meal and hot food, were adjusted to fit BKMR models. 

We conducted a sensitivity analysis in exploring the relationship between DII (as a continuous variable and a categorical variable) and ESCC utilizing unconditional logistic regression and restricted cubic splines (RCS) to evaluate the whole effect containing countervailing influences of dietary nutrients in the opposite directions of inflammation by a simple indicator. We implemented three sets of models: (1) unadjusted any covariates; (2) adjusted for gender and age; (3) the fully adjusted model, adjusting for gender, age, education, income, occupation, tobacco smoking, drinking intensity, tea consumption, eating speed per meal and hot food.

All statistical analyses were performed using the “compareGroups”, “glm”, “glmnet”, “rms” and “bkmr” packages of R software (R Core Team, 2022, Vienna, Austria; version 4.1.3), and all p values were based on two-sided tests.

## 3. Results

The baseline characteristics of the study subjects were listed and compared (Table 1). A total of 1064 participants were included in the analysis, consisting of 532 cases and 532 controls as sex- and age-frequency matches. Significant differences were observed between the two groups on daily energy intake, education, income, occupation, drinking intensity, tobacco smoking, tea consumption, eating speed per meal, hot food and DII (as a continuous and categorical variable) (all *p* values < 0.05). Compared with controls, ESCC patients were more likely to have statistically higher proportions of farmers and workers, lower education and income level, more severe degree of alcohol consumption, more frequently smoking tobacco and consuming tea, and poorer eating habits presented as quickly eating per meal and usually hot food. 

The distribution of nutrients between ESCC cases and the control group was represented (Table 2). In addition to cholesterol, selenium, thiamine, niacin, Vitamin E and PUFA, statistical differences were revealed in other nutrients between the two groups (all *p* value < 0.05).

The ESCC risk score was calculated through a linear combination of weighted coefficients for 22 nutrients with a generated coefficient-distribution map (Figure 2A). The cross-validation error graph in the most regularized and reduced model with coefficient paths differed in disparate logarithmic transformation lambda values (Figure 2B). Within one standard error of the minimum value, 13 nutrients eventually brought into BKMR analysis were protein, fat, carbohydrate, fiber, Vitamin A, riboflavin, niacin, Vitamin C, Fe, Se, MUFA, n-3 PUFA and n-6 PUFA. As listed in Table 2, these predictors were divided into two groups with properties of pro- or anti- [3 pro-inflammatory nutrients: protein, fat, and carbohydrate; 10 anti-inflammatory nutrients: fiber, Vitamin A, riboflavin, niacin, Vitamin C, Fe, Se, MUFA, n-3 PUFA and n-6 PUFA]. 

**Figure 3:**
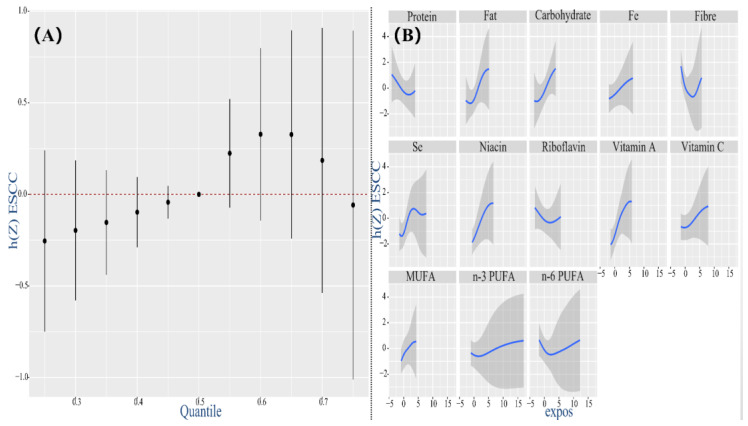
Associations between 13 dietary nutrients (predictors) and ESCC (outcome) by BKMR model adjusting for age, gender, education, income, occupation, tobacco smoking, drinking intensity, tea consumption, eating speed per meal and hot food. (**A**) The estimated overall effect of 13 dietary nutrients as compared to when all other nutrients are at the 50th percentile. The red dotted line means the reference level at which all of nutrients are fixed to their 50th percentile, to achieve interests of computing the overall effect of the mixture. (**B**) The univariate exposure–response function of each nutrient when setting the remaining nutrients at their median level. Abbreviation: BKMR, Bayesian kernel machine regression. Whereas the overall effect of whole nutrients tended to be pro−inflammatory, it was inappropriate to take all pro−inflammatory and anti−inflammatory predictors at the same time into account since there might be an offset influence. Hence, we further investigated respective effects and possible relationships with ESCC for each subset’s pro− and anti−inflammatory nutrients.

The full BKMR model, concurrently containing 13 nutrients with diverse inflammatory potential, revealed that the continuous markers of ESCC had an increasing trend even though there was no statistical difference when all nutrients were at their 60th and above percentile compared with the median level (Figure 3A). Although the overall effect was non-significant, individual nutrients displayed a certain non-linear correlation with the hazard of ESCC (Figure 3B). When all other nutrients were at the median level, n-3 PUFA, n-6 PUFA, protein, riboflavin, and fiber had a gradual or negative relationship with ESCC. In contrast, other nutrients represented by fat and carbohydrate were positively correlated with ESCC. Meanwhile, the single-exposure effects of individual nutrients on ESCC indicated that fat, carbohydrate and fiber in nutrient mixtures had significantly contributed to changes in ESCC (Figure S1A). Further exploring whether there was an interaction between nutrients, it set out mutual effects between fiber and fat, carbohydrate and Vitamin A, fiber and MUFA and MUFA and Vitamin A (Figure S1B). 

**Figure 4:**
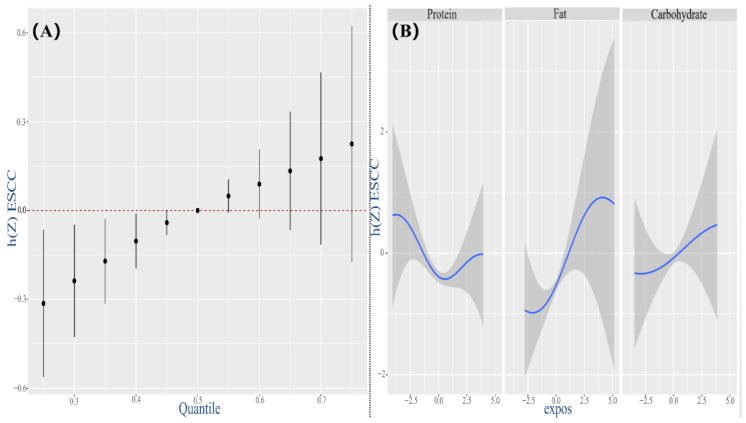
Associations between three pro−inflammatory nutrients (predictors) and ESCC (outcome) by BKMR model adjusting for age, gender, education, income, occupation, tobacco smoking, drinking intensity, tea consumption, eating speed per meal and hot food. (**A**) The estimated overall effect of 13 dietary nutrients as compared to when all other nutrients are at the 50th percentile. The red dotted line means the reference level at which all of nutrients are fixed to their 50th percentile, to achieve interests of computing the overall effect of the mixture. (**B**) The univariate exposure–response function of each nutrient when setting the remaining nutrients at their median level. Abbreviation: BKMR, Bayesian kernel machine regression.

**Figure 5:**
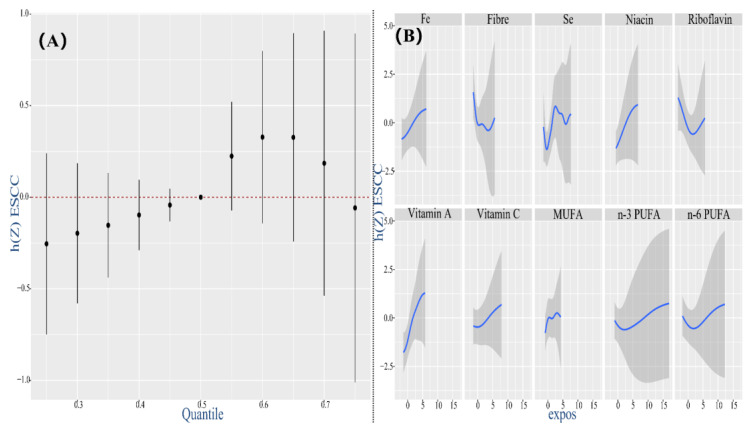
Associations between 10 anti−inflammatory nutrients (predictors) and ESCC (outcome) by BKMR model adjusting for age, gender, education, income, occupation, tobacco smoking, drinking intensity, tea consumption, eating speed per meal and hot food. (**A**) The estimated overall effect of 13 dietary nutrients as compared to when all of the other nutrients are at the 50th percentile. The red dotted line means the reference level at which all of nutrients are fixed to their 50th percentile, to achieve interests of computing the overall effect of the mixture. (**B**) The univariate exposure–response function of each nutrient when setting the remaining nutrients at their median level. Abbreviation: BKMR, Bayesian kernel machine regression. Even though results on univariate effects came to the same conclusion, the analysis comprising of collective nutrients in general revealed overall effect was a non−statistically risk factor for ESCC and was significantly distinct from that of the pro−inflammatory group, signifying a countervailing effect between pro−inflammatory and anti−inflammatory diets.

In the submodel only inclusive of three pro-inflammatory nutrients, when all pro-inflammatory nutrients were at or below their 40th percentile level, the potential continuity markers of ESCC risk revealed a significant increment compared with the median level, indicating that the combined effect of pro-inflammatory nutrients’ co-exposure was significantly positively correlated with ESCC (Figure 4A). In the anti-inflammatory nutrient model, a non-statistically significant difference was observed, but there was a downward trend (Figure 5A). 

When all other pro-inflammatory nutrients were fixed at median levels, fat and carbohydrate exhibited ascending association with ESCC risk, with a decrease trend for fat while an increasing trend for protein, in the high concentration (Figure 4B). Contemporaneously, in the anti-inflammatory nutrient model, fiber, riboflavin, n-3 PUFA and n-6 PUFA exhibited descending associations, with slight augment for the latent variables of ESCC at high concentrations. Fe, Se, niacin, Vitamin A, Vitamin C and MUFA depicted contrary results (Figure 5B). 

When observing single-predictor health risks for the pro-inflammatory nutrients’ subsets (Figure S2A) with other pro-inflammatory nutrients fixed at 25th, 50th and 75th percentiles, fats were significantly associated with gaining ESCC risk, although this relationship was attenuated at the 75th percentile of fat concentrations compared to the 25th percentile. On the contrary, for fiber and riboflavin, it delineated a significant and negative effect on the risk of ESCC when all of the other anti-inflammatory nutrients were fixed at their 50th and 75th percentiles (Figure S3A). 

The pro-inflammatory nutrients’ model disclosed interactions between protein and fat or carbohydrate, specifically that the estimated effect of fat or carbohydrate on ESCC risk was stronger when protein was set at lower concentrations (Figure S2B). Considering dissimilar slopes of one nutrient in different quantiles of the other between the two nutrient predictors, some potential interactions between MUFA and fiber, MUFA and Vitamin A, Se and niacin and Se and Vitamin A were observed in the anti-inflammatory nutrients’ model (Figure S3B).

**Figure 6:**
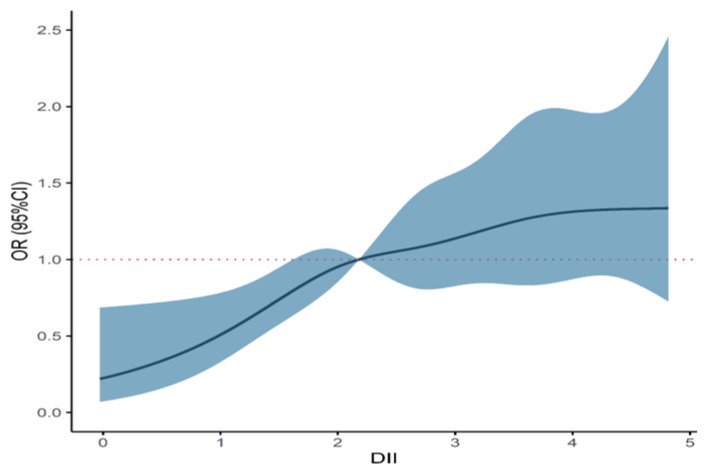
Restricted cubic spline curve fitting the relationship between DII and the risk of esophageal squamous cell cancer adjusting for age, gender, education, income, occupation, tobacco smoking, drinking intensity, tea consumption, eating speed per meal and hot food. The red dotted line stands for the reference level. The solid black line represents the OR, and the shaded part the lower and upper 95% CI; P_overall_ < 0.001; P_nonlinear_ < 0.001. Abbreviation: DII, dietary inflammatory index.

In our study, DII scores ranged between −0.864 and 4.891. The continuous scores were transformed into quartiles in light with its distribution among controls: Quartile 1 was the most anti-inflammatory (−0.864 to 1.727), and Quartile 4 was the most pro-inflammatory (4.159 to 4.891). In addition, the unconditional logistic model illustrated that a higher level of DII was significantly associated with ESCC (P_trend_ < 0.001), regardless of whether DII was considered as a continuous variable or a categorical variable (Table 3). Compared with the lowest quartile, the odds of ESCC for DII quartile four was 2.591 times (95% CI: 1.690, 3.971). The adjusted *OR* were 1.388 (95% CI: 1.206, 1.597) and 1.280 (95% CI: 1.118, 1.465) per 1-unit increase and per one-quartile increase, respectively. A similar trend result was graphically exhibited in RCS analysis (P_overall_ < 0.001; P_nonlinear_ < 0.001), indicating there was a positively non-linear relationship between inflammatory diets and ESCC risk (Figure 6). 

## 4. Discussion

This study explored the (1) the relationship between individual inflammatory nutrients and the risk of ESCC; (2) the overall impact of inflammatory nutrients and (3) the interaction between the different inflammatory nutrients. Thirteen nutrients were selected by LASSO regression, including three pro-inflammatory nutrients (protein, fat and carbohydrate) and ten anti-inflammatory nutrients (fiber, Vitamin A, riboflavin, niacin, Vitamin C, Fe, Se, MUFA, n-3 PUFA and n-6 PUFA). In the BKMR model, we pointed out that the overall effect of pro-inflammatory nutrients’ co-exposure was remarkably positively correlated with ESCC risks. Univariate exposure–response function and single-predictor health-risk analysis revealed ESCC patients tended to possess a higher level of fat and carbohydrate, while a lower level of fiber. Some interactions among nutrients were observed, for instance, fat or carbohydrate and protein, fiber and fat, carbohydrate and Vitamin A, etc. Sensitivity analysis indicated DII was positively and nonlinearly associated with risk of ESCC, which was in accordance with the results of the BKMR model. 

In line with our findings and postulations, it is well-reported that inflammation is engaged in the pathogenesis of many degenerative diseases including gastrointestinal cancer. In a study where a mice model [26] mimicked the development of human ESCC, it was identified that the down-regulation of CD8+ was accompanied by an immune response transformation that produced a chronic inflammatory environment and promoted the proliferation of carcinogen-transformed epithelial cells. Recently, cumulative scientific interests have posed that various abnormal changes in metabolic profile participated in the occurrence and development of ESCC through inflammatory factors and effects. Due to intrinsic antitumor properties, dihydroartemisinin inhibited ESCC by triggering cell pyroptosis [27], a novel pro-inflammatory programmed cell death. Pyroptosis-based cells exhibited the activation of caspase-8/3, the release of inflammatory factors (IL-18, IL-1β, etc.) and a strong relationship to inflammation, immunity and cancer. 

In the subgroup analysis that merely included dietary parameters with pro-inflammatory potential, fat was identified as a significant risk factor for ESCC. Similar to our results, some population-based studies stated that dietary intake of fat was associated with breast cancer [28], liver cancer [29] and colon cancer [30]. Likewise, relevant studies set forth that excessive dietary fat reservoir elicited a dysfunction of cellular process and activation of pro-inflammatory pathways, through unbalanced changes in gut microbiota and unfavorable remodeling in metabolic profiling, presented as fecal enrichment of arachidonic acids, anomalous lipopolysaccharide biosynthesis pathway and growth of plasma pro-inflammatory factors [31,32]. However, it was noteworthy that growing fats were thought to reduce ESCC risks when other predictors were fixed at the 25th, 50th and 75th percentile in the subset of pro-inflammatory nutrients. The mutual effect between fat and protein, obtained from bivariate exposure–response function analysis, might be a recipe for the paradoxical trend, implying further inquiry and elaboration. Corresponding to our results, a sub-study of the Protein Overfeeding trial [33] examined the changes in blood lipids under over-ingestion of high and low protein diets, elucidating that protein intake and fat intake were inversely related. Moreover, A prospective study [34] indicated that the association between higher take of dietary fat and gestational diabetes mellitus risk was drastically attenuated after adjustment for animal protein intake. It was not unique but had its counterpart. Composite effects of dietary protein and fat on several lipid parameters were ascertained [35]. In addition, we held that whether in the full parameter group or the anti-inflammatory parameter subgroup, fiber was thought to be a protective factor to reduce ESCC risk, in which there was a certain interaction between fiber and fat. Compatible with our opinions, the increases in dietary fat and fiber concentration were found to accelerate fecal excretion of bile acid (BA) with tumor promoting activity (*p* value < 0.05), which might change the specific binding of taurine and/or glycine in the liver and affect the lipid digestion/metabolism in the small intestine [36]. Diets high in fat and low in fiber contributed to metabolic endotoxemia, implicated as a cause of inflammation during metabolic dysfunction through changes in the gastrointestinal microbiome, bacterial fermentation end products, gut barrier function and enterohepatic circulation of BAs [37].

Considering that the interaction between dietary inflammatory nutrients might have a possible impact on ESCC risk, our study concluded that in the pro-inflammatory parameter subgroup, protein interacted not only with fat but also with carbohydrates. In accordance with our result, certain effects of carbohydrate and protein fermentation on gastrointestinal health were well established [38,39,40]. Diets with high protein and reduced carbohydrate altered the colonic tumor microenvironments, favoring a potentially pro-inflammatory microbiota profile and decreased short-chain fatty acid production. These aberrant changes largely compromised the colonic epithelium structure, causing mucosal inflammation that might also directly modulate the enteric nervous system and intestinal motility. Besides, the interaction between dietary components was found to regulate the transcription/translation process of lipid and carbohydrate metabolism genes via the activity of the protein kinase RNA-like endoplasmic reticulum stress response [41]. Furthermore, this study also noted that carbohydrate was considered to promote ESCC risk and interacted with Vitamin A in the full parameter group (including both pro-inflammatory and anti-inflammatory nutrients). It was reported that FGF19, a gut-derived hormone, controlled carbohydrate metabolism and was regulated by Vitamin A through farnesoid X receptor(FXR)-independent and -dependent pathways in human intestinal cell lines [42]. Interestingly, FXR synthetic ligand(GW4064) was thought to suppress ESCC proliferation through induction of apoptosis, arrest of cell cycle, inhibition of inflammatory genes and a reduction of ERK1/2 phosphorylation levels [43]. It suggests that FXR was somewhat associated with EESCC. The FXR-related comprehensive effect between vitamin A and carbohydrate has not been clarified, and further study is needed.

It is well-known that DII is broadly applied in fields of public health. As of yet, bulks of studies mainly concentrated on the relationship between DII and health outcomes in various gastrointestinal cancers, such as ESCC [18], colorectal cancer [19] and liver cancer [20]. To be compared with conventional studies, we also assessed the association of DII, as a synthetic index of nutrients, with ESCC risk. Similarly, we also noted DII was significantly associated with risks of ESCC in a positive and non-linear manner, signifying the pro-inflammatory diet was related to a higher risk of ESCC, which was consistent with the results of BKMR models. However, the effects of DII on ESCC risk haven’t been stated explicitly according to the single influence and the interactions among nutrients, which were sufficiently displayed in BKMR models from distinct perspectives of pro-inflammatory, anti-inflammatory and full nutrients. To sum up, DII, as a composite indicator, can efficiently assess the overall effect of nutrients with different inflammatory potency. Noteworthily, in addition to the explanation of the whole effects of all nutrients, this study based on the function of DII has the superiority in graphically and specifically revealing individual effects and mutual effects among nutrients that may be very important mechanistically. This implied that the relationship between dietary nutrients and ESCC may not be simply predicted by DII, and the exploration of the interaction and other effects of nutrients by BKMR can be used as a favorable supplement to DII so as to more holistically evaluate the association between inflammatory diets and ESCC [15,16,17].

The current study has several strengths. It is a newer analysis to look into, meticulously and comprehensively, the correlation between inflammation-related dietary nutrients and ESCC risks in the Chinese population from the perspective of inflammatory effects. Furthermore, LASSO regression, a shrinkage (penalized regression) method that pushed minimums of coefficients to exactly zero via directly shrinking the sum of the absolute values of coefficients, was robust enough to identify important mixture components (highly correlated variables) and address multicollinearity with lower coefficient variance than ordinary least-squares regression. Thirdly, the BKMR algorithm could synthetically identify non-linear exposure–reaction effects and graphically capture interactions between highly correlated nutrients’ mixed co-exposure, making up for sole usage of DII, allowing evaluation of combined effects of predictors with diverse influence directions. Finally, this study was conducted in multiple medical centers, which may reduce the potential selection bias to a certain extent. 

There are a few limitations in this study. The present study centering on the population in the southeast coastal area of China may be deficient in extrapolation. Additionally, one weakness of the BKMR model originated from its core algorithm, which fixed other inflammatory nutrients as predictors at a certain exposure level to infer the studied predictor’s exposure–response function and cannot estimate with advantage the effects of simultaneous exposure to high or low levels of food parameters.

## 5. Conclusions

Our study suggested that a pro-inflammatory diet increased ESCC risk while an anti-inflammatory diet reduced ESCC risk, implying that dietary nutrients and their inflammatory traits significantly impacted ESCC occurrence. Further research is warranted to validate these results and explore the probable pathobiological mechanisms, providing handholds for nutritional assessment and promoting early identification of ESCC.

## Figures and Tables

**Table 1:** Baseline Demographic Characteristics of the Analyzed Participants Given [Median (IQR) or Frequency (%)].

Variables	Control (N = 532)	ESCC (N = 532)	*p* Value
Age			0.950 ^a^
<60	319 (60.0)	318 (59.8)	
≥60	213 (40.0)	214 (40.2)	
Gender			0.790 ^a^
Male	369 (69.4)	373 (70.1)	
Female	163 (30.6)	159 (29.9)	
Daily energy intake (Kcal/day)	1363 [943.0;1942.0]	996 [761.0;1356.0]	<0.001 ^b^
Education level			<0.001 ^a^
Illiteracy/Primary school	234 (44.0)	332 (62.4)	
Junior high school/above	298 (56.0)	200 (37.6)	
Income level			<0.001 ^a^
<¥2000/month	154 (28.9)	217 (40.8)	
¥2000–5000/month	254 (47.7)	219 (41.2)	
>¥5000/month	124 (23.3)	96 (18.0)	
Occupation			<0.001 ^a^
Farmer	155 (29.1)	229 (43.0)	
Worker	68 (12.8)	103 (19.4)	
Individual Business	89 (16.7)	56 (10.5)	
Other	220 (41.4)	144 (27.1)	
Tobacco smoking			<0.001 ^a^
No	311 (58.5)	217 (40.8)	
Yes	221 (41.5)	315 (59.2)	
Drinking intensity			<0.001 ^a^
No	240 (45.1%)	230 (43.2%)	
Mild	157 (29.5%)	63 (11.8%)	
Moderate	87 (16.4%)	155 (29.1%)	
Heavy	48 (9.0%)	82 (15.4%)	
Tea consumption			<0.001 ^a^
Never/seldom	308 (57.9)	239 (44.9)	
Often	224 (42.1)	293 (55.1)	
Eating speed/each meal			0.002 ^a^
Normal (10–20 min)	286 (53.8)	242 (45.5)	
Quick (<10 min)	123 (23.1)	173 (32.5)	
Slow (>20 min)	123 (23.1)	117 (22.0)	
Hot food			<0.001 ^a^
No	173 (32.5)	174 (32.7)	
Occasionally	272 (51.1)	161 (30.3)	
Often	87 (16.4)	197 (37.0)	
DII (continuous)	2.38 [1.50;3.43]	3.05 [2.25;3.89]	<0.001 ^b^
DII (category)			<0.001 ^a^
Quartile 1	133 (25.0)	66 (12.4)	
Quartile 2	133 (25.0)	100 (18.8)	
Quartile 3	133 (25.0)	168 (31.6)	
Quartile 4	133 (25.0)	198 (37.2)	

^a^ χ^2^ test between cases and controls; ^b^ Mann–Whitney U test between cases and controls; Abbreviation: DII, dietary inflammation index.

**Table 2:** Distribution of Nutrient Content Specific for DII in 1064 Subjects among Cases and Controls [Median (IQR)].

Characteristic	Control (N = 532)	ESCC (N = 532)	*p* Value ^a^	Enter in BKMR Model ^b^
Pro-inflammatory				
Protein (g/day)	51.6 [45.9; 57.8]	48.8 [40.9; 55.8]	<0.001	Yes
Total fat (g/day)	38.8 [32.3; 46.0]	44.0 [35.1; 52.7]	<0.001	Yes
Carbohydrate (g/day)	109.0 [92.2; 130.0]	102.0 [82.9; 124.0]	<0.001	Yes
Cholesterol (mg/day)	319.0 [211.0; 447.0]	304.0 [185.0; 456.0]	0.216	No
Ferrum (mg/day)	15.1 [12.1; 19.4]	13.8 [10.9; 18.0]	<0.001	Yes
Saturated fat (g/day)	17.3 [11.5; 208.0]	68.2 [13.5; 221.0]	<0.001	No
Anti-inflammatory				
Fiber (g/day)	8.1 [5.8; 12.2]	5.9 [4.0; 8.7]	<0.001	Yes
Zinc (mg/day)	7.4 [6.6; 8.6]	7.2 [5.9; 8.4]	0.003	No
Selenium (μg/day)	37.8 [30.4; 51.4]	35.8 [24.8; 55.8]	0.027	Yes
Magnesium (mg/day)	237.0 [192.0; 291.0]	208.0 [165.0; 264.0]	<0.001	No
Vitamin A (RE/day)	442.0 [314.0; 615.0]	387.0 [268.0; 576.0]	0.001	Yes
β-Carotene (μg/day)	2956.0 [1504.0; 4993.0]	2426.0 [1412.0; 4323.0]	0.009	No
Thiamine (mg/day)	0.6 [0.5; 0.7]	0.6 [0.5; 0.8]	0.113	No
Riboflavin (mg/day)	0.8 [0.7; 0.9]	0.7 [0.5; 0.8]	<0.001	Yes
Niacin (mg/day)	13.6 [10.8; 121.0]	12.8 [9.9; 200.0]	0.337	Yes
Vitamin C (mg/day)	96.5 [65.4; 143.0]	82.5 [50.9; 128.0]	<0.001	Yes
Vitamin E (mg/day)	12.1 [7.7; 17.8]	13.2 [8.34; 18.1]	0.099	No
Monounsaturated fat (g/day)	19.2 [12.7; 245.0]	78.3 [14.3; 261.0]	0.001	Yes
Polyunsaturated fats (g/day)	5.9 [4.0; 96.3]	33.6 [4.1; 95.6]	0.230	No
N-3 polyunsaturated fatty acid (g/day)	2.8 [1.9; 3.9]	2.5 [1.7; 3.8]	0.017	Yes
N-6 polyunsaturated fatty acid (g/day)	20.8 [16.0; 26.8]	19.2 [13.9; 25.7]	0.001	Yes
Alcohol (g/day)	0.00 [0.00; 5.93]	0.90 [0.00; 30.0]	<0.001	No

^a^ Mann–Whitney U test for the difference between cases and controls; ^b^ The nutrient selection using LASSO regression with ten-fold cross-validation and one standard error of the minimum logarithmic transformation lambda value to judge whether to enter into the BKMR model. Abbreviation: LASSO, least absolute shrinkage and selection operator; BKMR, Bayesian kernel machine regression.

**Table 3:** Adjusted Odds Ratios (OR) and 95% Confidence Intervals (CI) for the Association Between DII and Esophageal Squamous Cell Cancer.

Variable	*OR*(95% CI)		
	Model 1	Model 2 ^a^	Model 3 ^b^
Continuous, per 1-unit increase	1.490 (1.323–1.677)	1.470 (1.305–1.657)	1.388 (1.206–1.597)
Category			
Quartile 1 −0.864 to 1.727	1 (Reference)	1 (Reference)	1 (Reference)
Quartile 2 1.728 to 2.771	1.969 (1.330–2.914)	1.992 (1.343–2.954)	1.807 (1.167–2.797)
Quartile 3 2.772 to 4.158	2.872 (1.964–4.199)	2.735 (1.865–4.009)	2.175 (1.405–3.367)
Quartile 4 4.159 to 4.891	3.033 (2.078–4.427)	2.950 (2.018–4.312)	2.591 (1.690–3.971)
p for trend	<0.001	<0.001	<0.001
per one-quartile increase	1.396 (1.244–1.567)	1.369 (1.219–1.538)	1.280 (1.118–1.465)

^a^ Adjusted for age and gender, using unconditional logistic regression. ^b^ Adjusted for age, gender, education, income, occupation, tobacco smoking, drinking intensity, tea consumption, eating speed per meal and hot food, using unconditional logistic regression.

## Data Availability

The datasets used in this study are available from the corresponding author on reasonable request.

## Supplementary Materials

The following supporting information can be downloaded at: https://www.mdpi.com/article/10.3390/nu14235179/s1, Figure S1: Associations between 13 dietary nutrients(predictors) and ESCC(outcome) by BKMR model adjusting for age, gender, education, income, occupation, tobacco smoking, drinking intensity, tea consumption, eating speed per meal, and hot food; Figure S2: Associations between 3 pro-inflammatory nutrients(predictors) and ESCC(outcome) by BKMR model adjusting for age, gender, education, income, occupation, tobacco smoking, drinking intensity, tea consumption, eating speed per meal, and hot food; Figure S3: Associations between 10 anti-inflammatory nutrients(predictors) and ESCC(outcome) by BKMR model adjusting for age, gender, education, income, occupation, tobacco smoking, alcohol drinking intensity, tea consumption, eating speed per meal, and hot food.
