# Supplementary material for: Dietary Inflammatory Nutrients and Esophageal Squamous Cell Carcinoma Risk: A Case-Control Study
Source: Nutrients. 2022 Dec 5;14(23):5179. doi: 10.3390/nu14235179 (PMC9737973; doi:10.3390/nu14235179)
Supplement: Supplementary file 1 [file nutrients-14-05179-s001.zip › nutrients-2015955-supplementary.pdf]

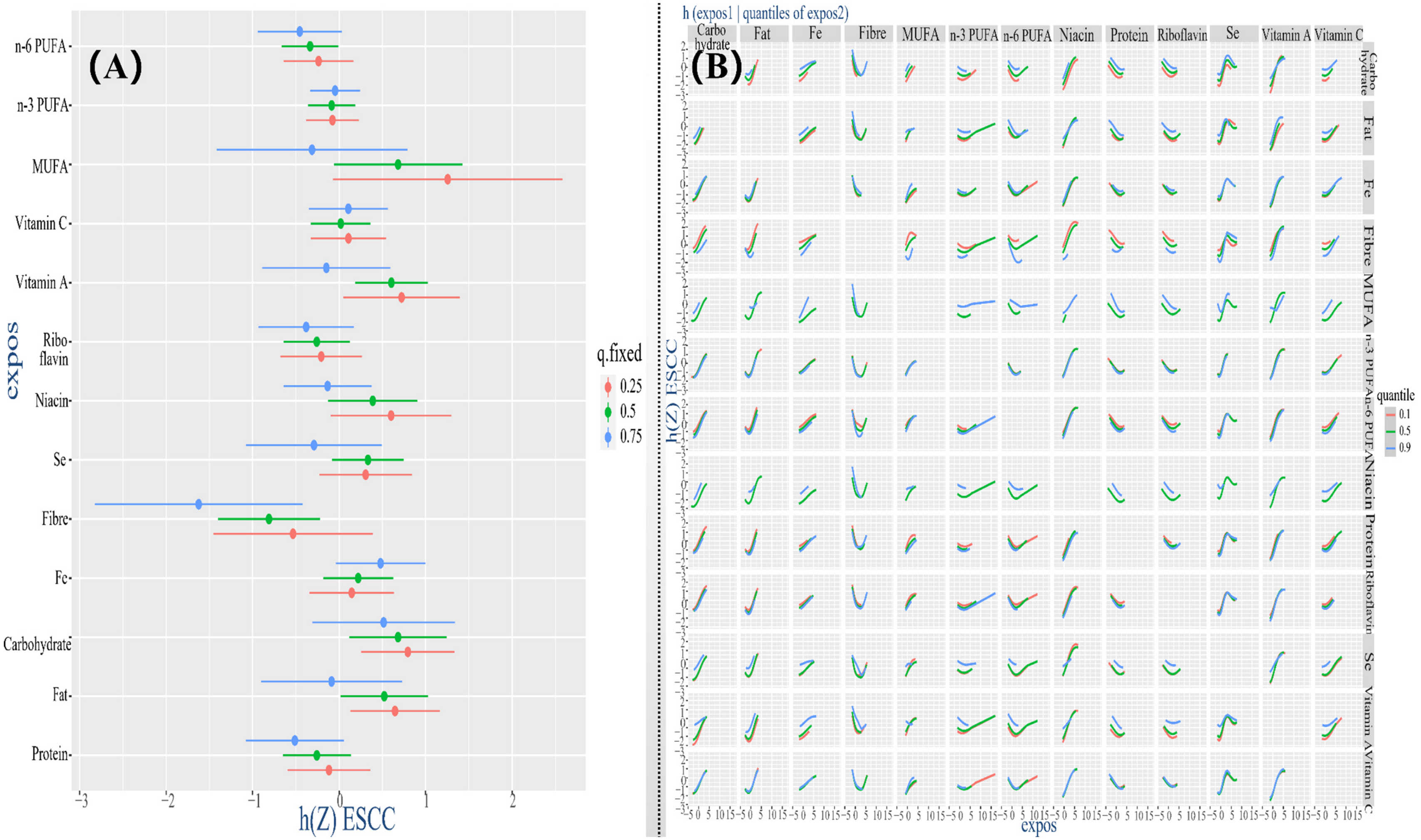

Figure S1. Associations between 13 dietary nutrients(predictors) and ESCC(outcome) by BKMR model adjusting for age, gender, education, income, occupation, tobacco smoking, drinking intensity, tea consumption, eating speed per meal, and hot food. (A) The single−predictor ESCC risks when all of the other nutrients are specified to their 75th percentile and to their 25th percentile. (B) The bivariate exposure−response function of each two nutrients in ESCC risk (a single nutrient for the second nutrient fixed at various quantiles), where all other predictors are fixed at a particular quantile(50th).

Abbreviation: BKMR, bayesian kernel machine regression.

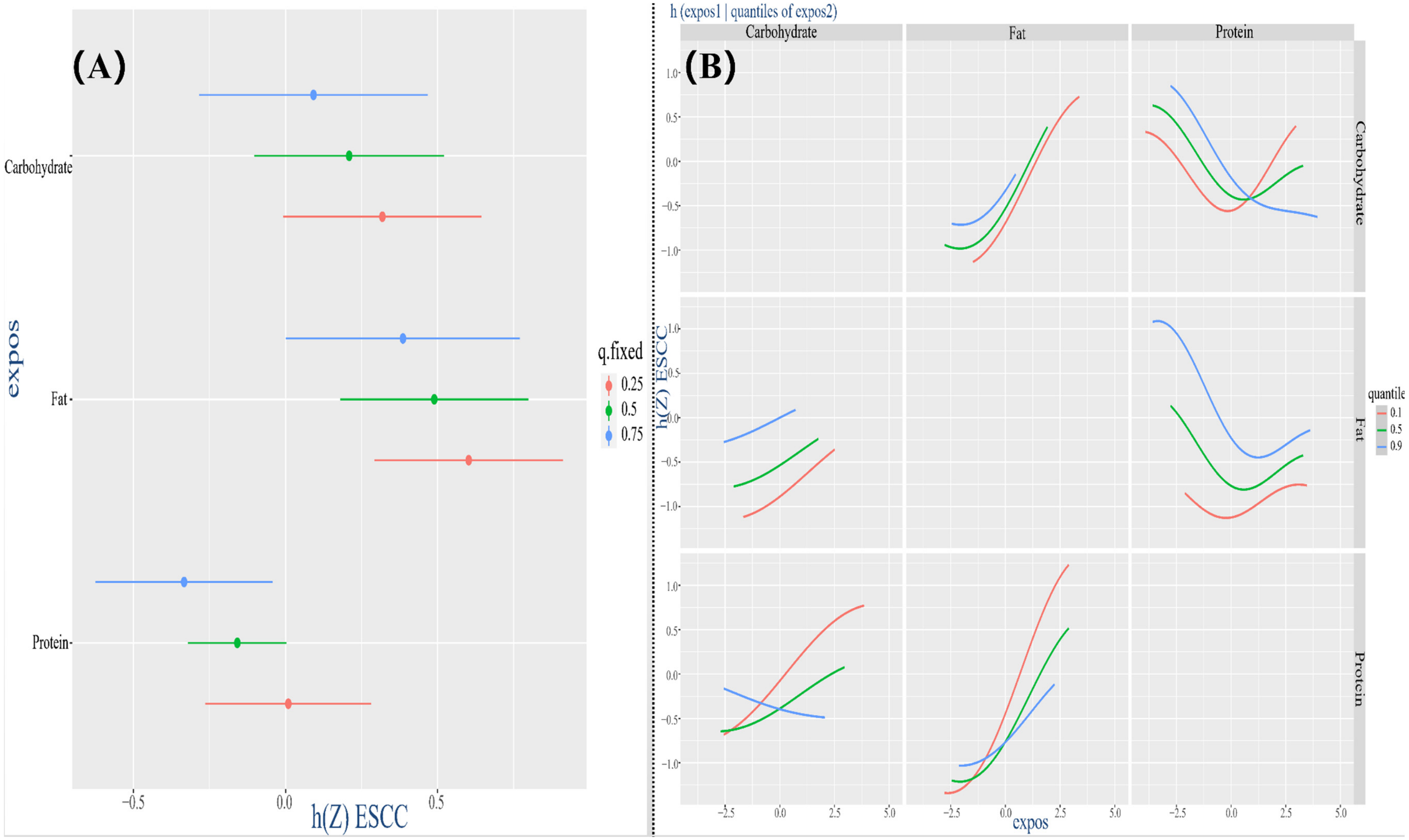

Figure S2. Associations between 3 pro−inflammatory nutrients(predictors) and ESCC(outcome) by BKMR model adjusting for age, gender, education, income, occupation, tobacco smoking, drinking intensity, tea consumption, eating speed per meal, and hot food. (A) The single−predictor ESCC risks when all of the other nutrients are specified to their 75th percentile and their 25th percentile. (B) The bivariate exposure−response function of each two nutrients in ESCC risk (a single nutrient for the second nutrient fixed at various quantiles), where all of the other predictors are fixed at a particular quantile(50th).

Abbreviation: BKMR, bayesian kernel machine regression.

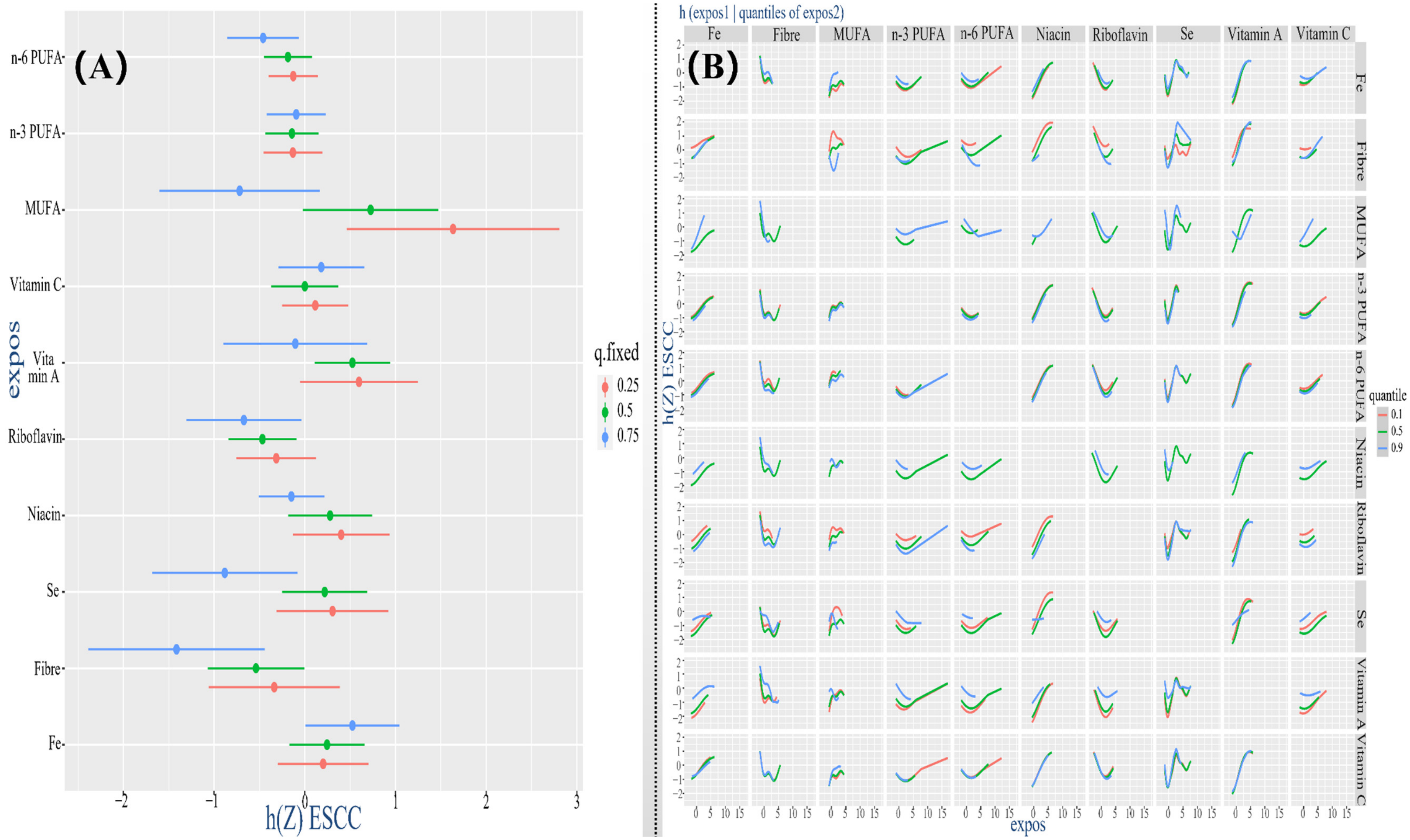

Figure S3. Associations between 10 anti−inflammatory nutrients(predictors) and ESCC(outcome) by BKMR model adjusting for age, gender, education, income, occupation, tobacco smoking, alcohol drinking intensity, tea consumption, eating speed per meal, and hot food. (A) The single−predictor ESCC risks when all other nutrients are specified to their 75th and 25th percentile. (B) The bivariate exposure−response function of each two nutrients in ESCC risk (a single nutrient for the second nutrient fixed at various quantiles), where all other predictors are fixed at a particular quantile(50th).

Abbreviation: BKMR, bayesian kernel machine regression.
